# The genome sequence of a ground beetle, *Nebria brevicollis* (Fabricius, 1792)

**DOI:** 10.12688/wellcomeopenres.18749.1

**Published:** 2023-01-12

**Authors:** Liam Crowley, University of Oxford and Wytham Woods Genome Acquisition Lab, Darwin Tree of Life Barcoding collective, Wellcome Sanger Institute Tree of Life programme, Wellcome Sanger Institute Scientific Operations: DNA Pipelines collective, Tree of Life Core Informatics collective, Beulah Garner, Darwin Tree of Life Consortium

**Affiliations:** 1 Department of Biology, University of Oxford, Oxford, UK; 2 Insects Division, Department of Life Sciences, Natural History Museum, London, UK

**Keywords:** Nebria brevicollis, ground beetle, genome sequence, chromosomal, Coleoptera

## Abstract

We present a genome assembly from an individual female *Nebria brevicollis* (a ground beetle; Arthropoda; Insecta; Coleoptera; Carabidae). The genome sequence is 242 megabases in span. Most of the assembly is scaffolded into 15 chromosomal pseudomolecules, with the X sex chromosome assembled. The mitochondrial genome has also been assembled and is 25.2 kilobases in length. Gene annotation of this assembly on Ensembl identified 11,021 protein-coding genes.

## Species taxonomy

Eukaryota; Metazoa; Ecdysozoa; Arthropoda; Hexapoda; Insecta; Pterygota; Neoptera; Endopterygota; Coleoptera; Adephaga; Caraboidea; Carabidae; Nebriinae; Nebriini; Nebria; *Nebria*; *Nebria brevicollis* (Fabricius, 1792) (NCBI:txid110024)

## Background

*Nebria brevicollis* (Coleoptera, Carabidae) is a relatively large ground beetle, measuring 10–14 mm, closely resembling other members of the Nebria Latreille, 1802 and Leistus Frölich, 1799 genera. *N. brevicollis* is distinguished from others by a uniformly dark brown or black habitus; always with completely brown antennae. Humeral angles of the elytra are discontinuous and with a small protruding tooth, though this tooth can sometimes appear more as simply distinctly angled. The basal abdominal sternite is punctured laterally ( Luff, 2007). It can be easily separated from its close relative *N. salina* by a fine pubescence of the dorsal surface of the hind tarsi, which are not present in *N. salina*.

*Nebria brevicollis* is a ubiquitous, widely distributed and commonly found beetle throughout the UK. It prefers the moister habitats of woodland, heathland, moorland and parkland, venturing into stable coastal (but not saline) areas. It can also be found in wasteland, industrial areas and gardens. It lives under rocks, logs and moist debris. Though native to temperate Europe, ranging through the Caucasus and Asia Minor, its range has expanded to North America, where it is considered an invasive species ( LaBonte, 2011).

This species is an opportunistic, omnivorous nocturnal predator. It has been recorded as feeding on Mollusca, earthworms, various arachnids, and other small insects, including other beetles, in both larval and imaginal stages of their lifecycles ( Larochelle, 1990).

This species can be found throughout the year but is most active from June to August. After an intensive feeding period they enter diapause, emerging to mate in the Autumn ( Penney, 1969). Larvae are winter active, with the first adults appearing in late winter to early spring.

The *Nebria* genus is amongst the ground beetles capable of surviving at high altitudes ( Gobbi, 2020). This ability, along with a eurytopic life-history, makes this genus of interest for climate-change studies. The genome sequence of the Nebriine described here is useful for investigations into species’ ability to withstand and adapt to depauperate environments, in a world of increasing climatic instability ( Butterfield, 1996), and its potential impact on native populations of other invertebrates as climate change and anthropogenic effects allow it to expand its ecological and geographical range.

### Genome sequence report

**Figure 1.**
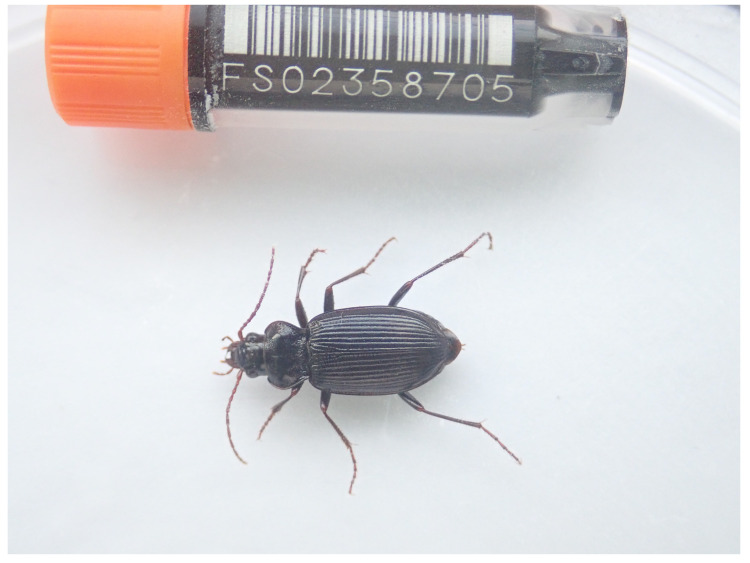
Image of the *Nebria brevicollis* (icNebBrev1) specimen used for genome sequencing.

The genome was sequenced from one female *Nebria brevicollis* specimen (icNebBrev1: Figure 1) collected from Wytham Woods (latitude 51.77, longitude –1.34). A total of 70-fold coverage in Pacific Biosciences single-molecule HiFi long reads and 76-fold coverage in 10X Genomics read clouds was generated. Primary assembly contigs were scaffolded with chromosome conformation Hi-C data. Manual assembly curation corrected 304 missing joins or mis-joins and removed three haplotypic duplications, reducing the scaffold number by 38.73%, and increasing the scaffold N50 by 246.17%.

**Figure 2.**
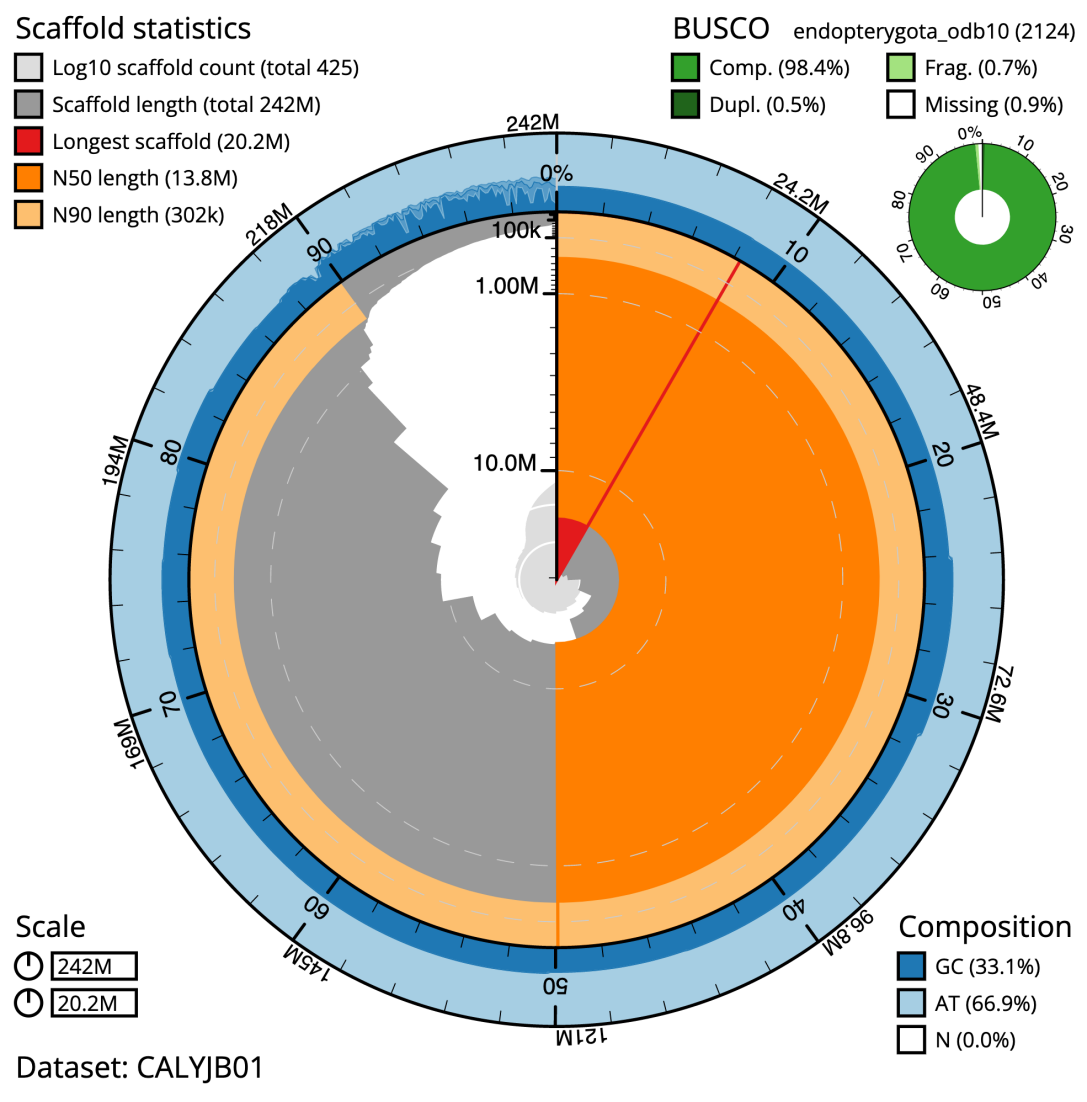
Genome assembly of *Nebria brevicollis*, icNebBrev1.1: metrics. The BlobToolKit Snailplot shows N50 metrics and BUSCO gene completeness. The main plot is divided into 1,000 size-ordered bins around the circumference with each bin representing 0.1% of the 241,934,194 bp assembly. The distribution of scaffold lengths is shown in dark grey with the plot radius scaled to the longest scaffold present in the assembly (20,209,999 bp, shown in red). Orange and pale-orange arcs show the N50 and N90 scaffold lengths (13,798,286 and 301,996 bp), respectively. The pale grey spiral shows the cumulative sequence count on a log scale with white scale lines showing successive orders of magnitude. The blue and pale-blue area around the outside of the plot shows the distribution of GC, AT and N percentages in the same bins as the inner plot. A summary of complete, fragmented, duplicated and missing BUSCO genes in the endopterygota_odb10 set is shown in the top right. An interactive version of this figure is available at https://blobtoolkit.genomehubs.org/view/icNebBrev1.1/dataset/CALYJB01/snail.

The final assembly has a total length of 241.9 Mb in 424 sequence scaffolds with a scaffold N50 of 13.8 Mb ( Table 1). Most (86.87%) of the assembly sequence was assigned to 15 chromosomal-level scaffolds, representing 14 autosomes and the X sex chromosome ( Figure 2– Figure 5; Table 2). Chromosome-scale scaffolds confirmed by the Hi-C data are named in order of size. The order and orientation of scaffolds within the centromeric region of chromosomes 1, 3, 4, 8 and 10 is uncertain ( Figure 5). The assembly has a BUSCO v5.3.2 ( Manni *et al.*, 2021) completeness of 98.4% (single 97.9%, duplicated 0.5%), using the OrthoDB v10 endopterygota reference set. While not fully phased, the assembly deposited is of one haplotype. Contigs corresponding to the second haplotype have also been deposited.

**Table 1.** Genome data for *Nebria brevicollis*, icNebBrev1.1.

Project accession data		
Assembly identifier	icNebBrev1.1	
Species	*Nebria brevicollis*	
Specimen	icNebBrev1	
NCBI taxonomy ID	110024	
BioProject	PRJEB49041	
BioSample ID	SAMEA7520209	
Isolate information		
Assembly metrics *		*Benchmark*
Consensus quality (QV)	48.8	*≥ 50*
*k*-mer completeness	99.95%	*≥ 95%*
BUSCO **	C: 98.4%[S: 97.9%,D: 0.5%],F: 0.7%,M: 0.9%,n: 2,124	*C ≥ 95%*
Percentage of assembly mapped to chromosomes	86,87%	*≥ 95%*
Sex chromosomes	X chromosome	*localised* *homologous* *pairs*
Organelles	Mitochondrial genome assembled	*complete* *single alleles*
Raw data accessions		
PacificBiosciences SEQUEL II	ERR8363510	
10X Genomics Illumina	ERR7440916–ERR7440919	
Hi-C Illumina	ERR7440920	
Genome assembly		
Assembly accession	GCA_944738965.1	
*Accession of alternate* *haplotype*	GCA_944739395.1	
Span (Mb)	241.9	
Number of contigs	787	
Contig N50 length (Mb)	2.5	
Number of scaffolds	424	
Scaffold N50 length (Mb)	13.8	
Longest scaffold (Mb)	20.2	
Genome annotation		
Number of protein-coding genes	11,021	
Non-coding genes	3,253	
Number of transcripts	20,797	

* Assembly metric benchmarks are adapted from column VGP-2020 of “ Table 1: Proposed standards and metrics for defining genome assembly quality” from ( Rhie *et al.*, 2021). ** BUSCO scores based on the endopterygota_odb10 BUSCO set using v5.3.2. C = complete [S = single copy, D = duplicated], F = fragmented, M = missing, n = number of orthologues in comparison. A full set of BUSCO scores is available at https://blobtoolkit.genomehubs.org/view/icNebBrev1.1/dataset/CALYJB01/busco.

**Figure 3.**
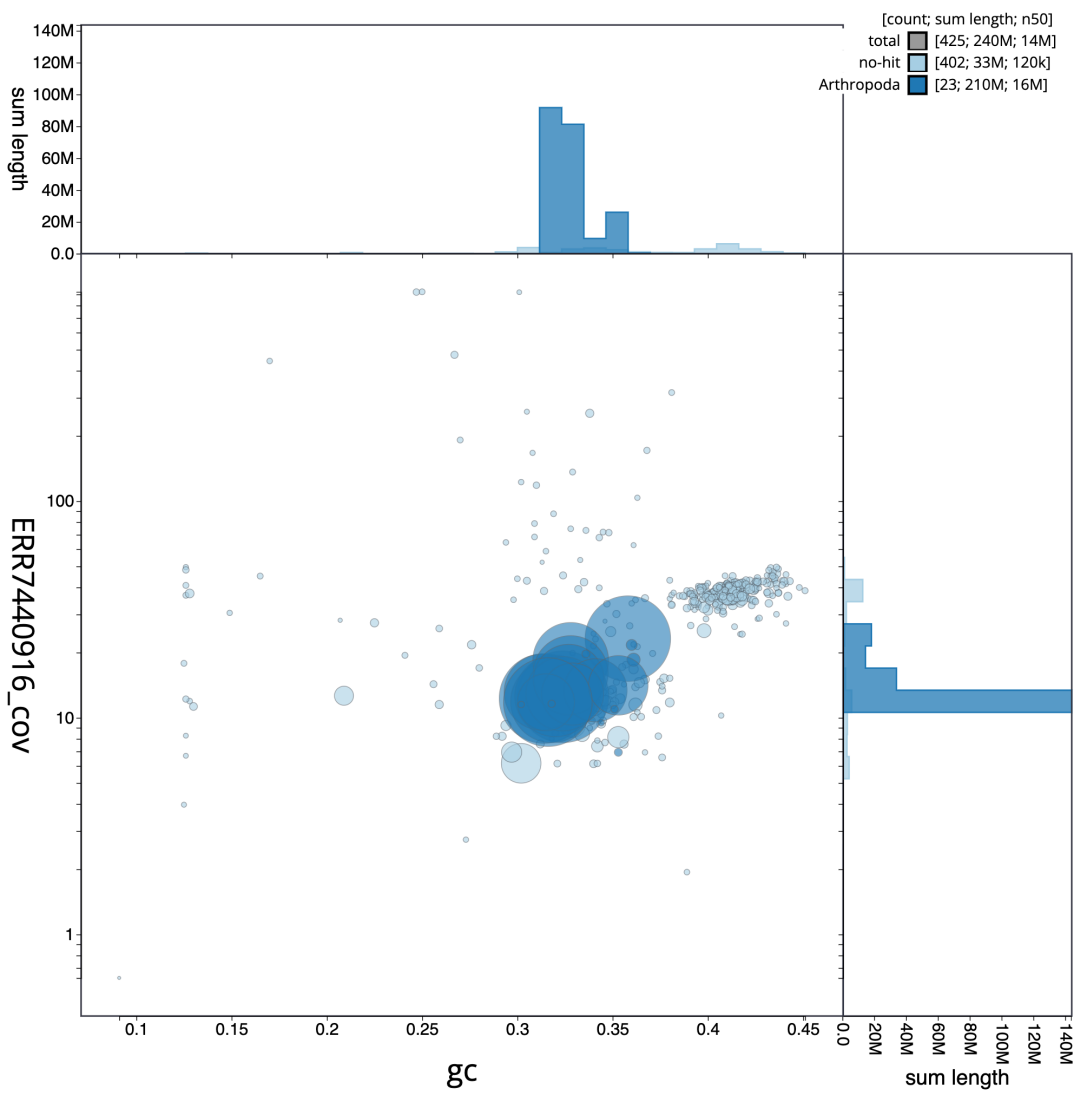
Genome assembly of *Nebria brevicollis*, icNebBrev1.1: GC coverage. BlobToolKit GC-coverage plot. Scaffolds are coloured by phylum. Circles are sized in proportion to scaffold length. Histograms show the distribution of scaffold length sum along each axis. An interactive version of this figure is available at https://blobtoolkit.genomehubs.org/view/icNebBrev1.1/dataset/CALYJB01/blob.

**Figure 4.**
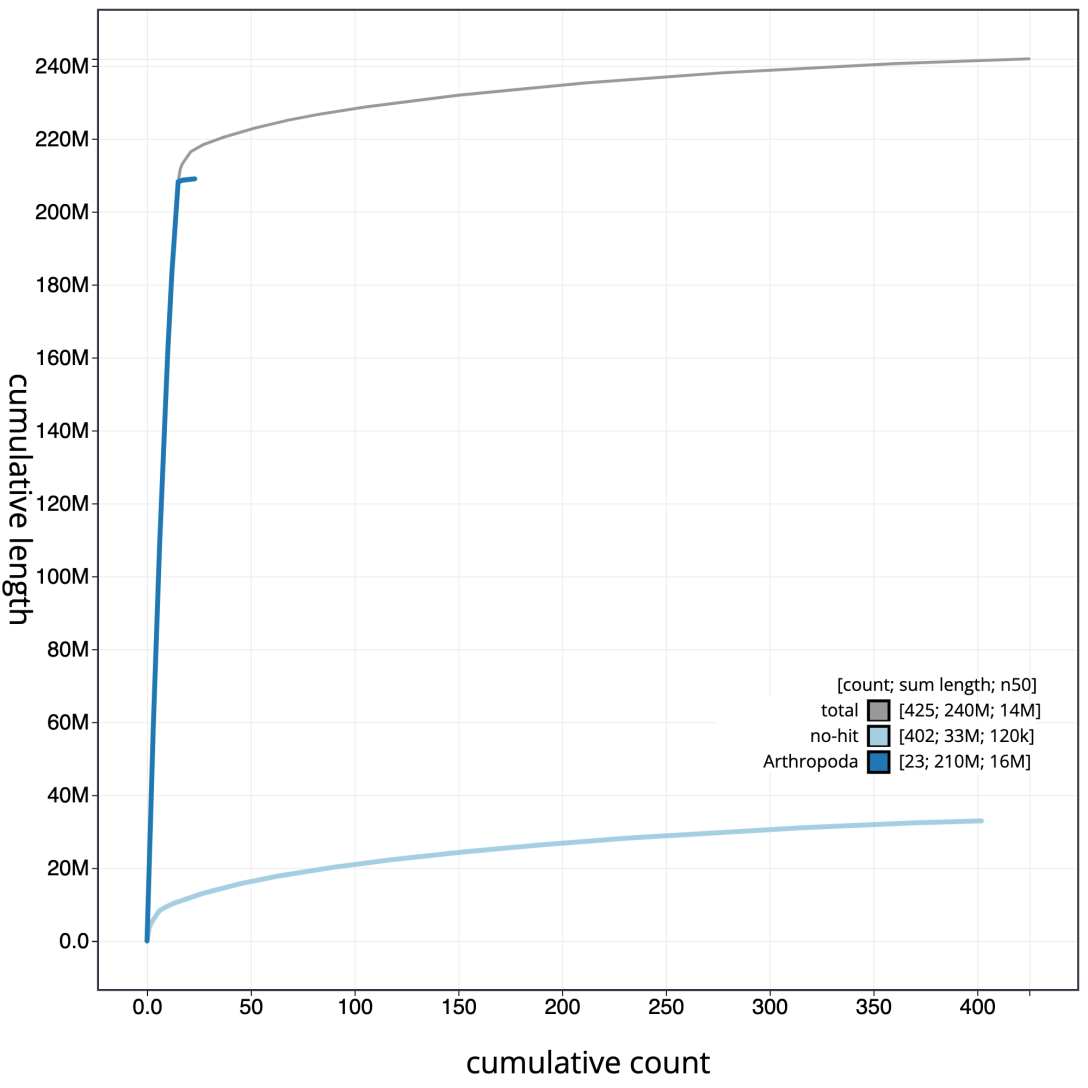
Genome assembly of *Nebria brevicollis*, icNebBrev1.1: cumulative sequence. BlobToolKit cumulative sequence plot. The grey line shows cumulative length for all scaffolds. Coloured lines show cumulative lengths of scaffolds assigned to each phylum using the buscogenes taxrule. An interactive version of this figure is available at https://blobtoolkit.genomehubs.org/view/icNebBrev1.1/dataset/CALYJB01/cumulative.

**Figure 5.**
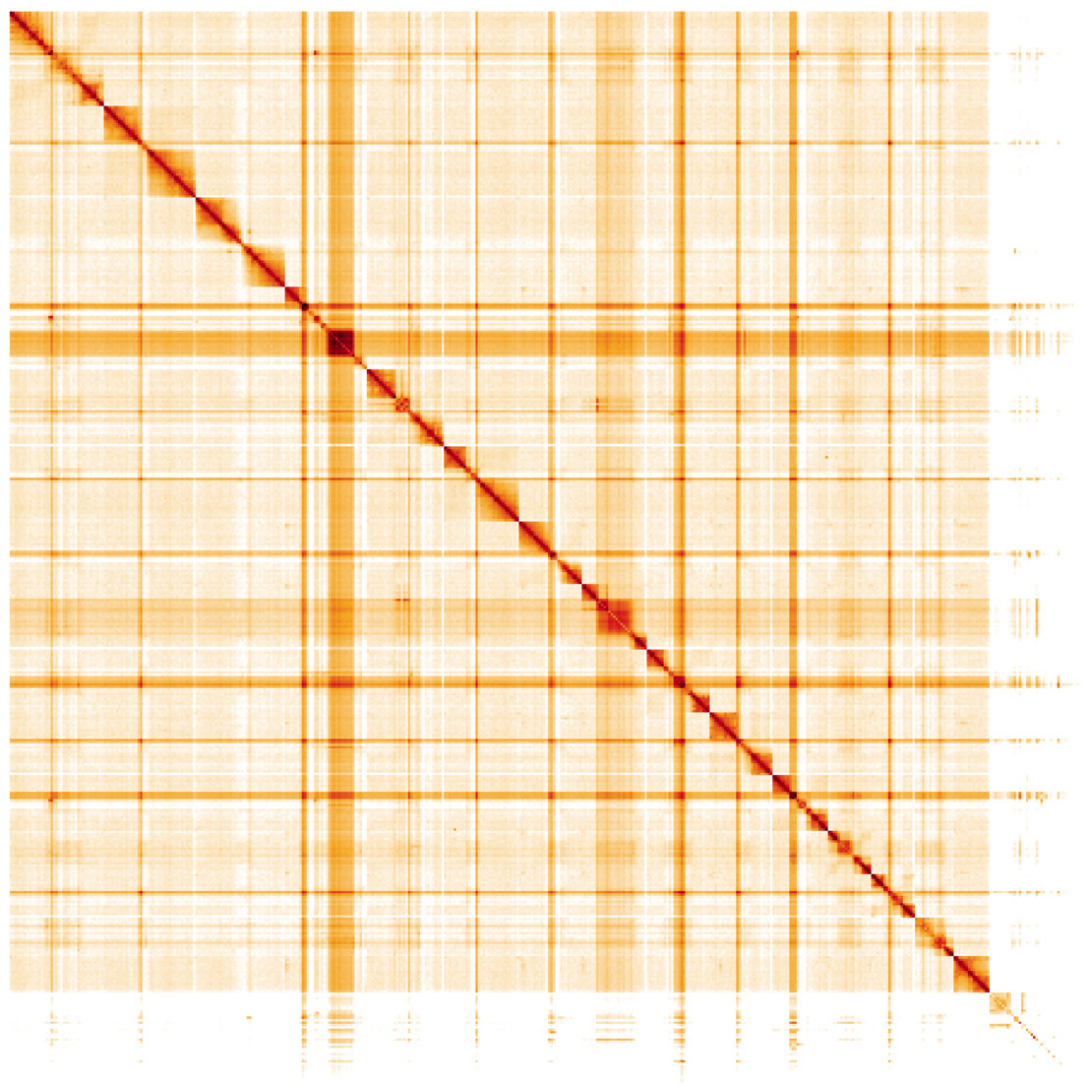
Genome assembly of *Nebria brevicollis*, icNebBrev1.1: Hi-C contact map. Hi-C contact map of the icNebBrev1.1 assembly, visualised using HiGlass. Chromosomes are shown in order of size from left to right and top to bottom. An interactive version of this figure may be viewed at https://genome-note-higlass.tol.sanger.ac.uk/l/?d=Uq6H4s_FTWuI6hhSy2sL3A.

**Table 2.** Chromosomal pseudomolecules in the genome assembly of *Nebria brevicollis*, icNebBrev1.

INSDC accession	Chromosome	Size (Mb)	GC%
OX122897.1	1	20.21	32.4
OX122898.1	2	19.37	31.4
OX122900.1	3	17.58	35.8
OX122901.1	4	16.14	31.9
OX122902.1	5	15.82	32
OX122903.1	6	13.8	31.3
OX122904.1	7	13.65	32.8
OX122905.1	8	13.38	32.8
OX122906.1	9	13.15	32.4
OX122907.1	10	11.82	32.7
OX122908.1	11	9.41	34.1
OX122909.1	12	9.06	32.9
OX122910.1	13	8.28	35.3
OX122911.1	14	7.59	31.5
OX122899.1	X	18.92	31.6
OX122912.1	MT	0.03	16.8
-	-	33.74	35.9

### Genome annotation report

The *N. brevicollis* genome assembly was annotated using the Ensembl rapid annotation pipeline ( Table 1; GCA_944738965.1). The resulting annotation includes 20,797 transcribed mRNAs from 11,021 protein-coding and 3,253 non-coding genes.

## Methods

### Sample acquisition and nucleic acid extraction

A female *Nebria brevicollis* (icNebBrev1) was collected in woodland habitat of the Great Wood, Wytham, Berkshire, UK (latitude 51.773, longitude –1.338) on 9 October 2019, using a pitfall trap. The specimen was collected and identified by Liam Crowley (University of Oxford), and preserved on dry ice using a CoolRack.

DNA was extracted at the Tree of Life laboratory, Wellcome Sanger Institute. The icNebBrev1 sample was weighed and dissected on dry ice with tissue set aside for Hi-C sequencing. Head and thorax tissue was disrupted using a Nippi Powermasher fitted with a BioMasher pestle. High molecular weight (HMW) DNA was extracted using the Qiagen MagAttract HMW DNA extraction kit. Low molecular weight DNA was removed from a 20 ng aliquot of extracted DNA using 0.8X AMpure XP purification kit prior to 10X Chromium sequencing; a minimum of 50 ng DNA was submitted for 10X sequencing. HMW DNA was sheared into an average fragment size of 12–20 kb in a Megaruptor 3 system with speed setting 30. Sheared DNA was purified by solid-phase reversible immobilisation using AMPure PB beads with a 1.8X ratio of beads to sample to remove the shorter fragments and concentrate the DNA sample. The concentration of the sheared and purified DNA was assessed using a Nanodrop spectrophotometer and Qubit Fluorometer and Qubit dsDNA High Sensitivity Assay kit. Fragment size distribution was evaluated by running the sample on the FemtoPulse system.

### Sequencing

Pacific Biosciences HiFi circular consensus and 10X Genomics read cloud DNA sequencing libraries were constructed according to the manufacturers’ instructions. DNA sequencing was performed by the Scientific Operations core at the WSI on Pacific Biosciences SEQUEL II (HiFi) and HiSeq X Ten (10X) instruments. Hi-C data were also generated from abdomen tissue of icNebBrev1 using the Arima v2 kit and sequenced on the Illumina NovaSeq 6000 instrument.

### Genome assembly

Assembly was carried out with Hifiasm ( Cheng *et al.*, 2021) and haplotypic duplication was identified and removed with purge_dups ( Guan *et al.*, 2020). One round of polishing was performed by aligning 10X Genomics read data to the assembly with Long Ranger ALIGN, calling variants with freebayes ( Garrison & Marth, 2012). The assembly was then scaffolded with Hi-C data ( Rao *et al.*, 2014) using YaHS ( Zhou *et al.*, 2022). The assembly was checked for contamination as described previously ( Howe *et al.*, 2021). Manual curation was performed using HiGlass ( Kerpedjiev *et al.*, 2018) and Pretext ( Harry, 2022). The mitochondrial genome was assembled using MitoHiFi ( Uliano-Silva *et al.*, 2021), which performed annotation using MitoFinder ( Allio *et al.*, 2020). The genome was analysed and BUSCO scores generated within the BlobToolKit environment ( Challis *et al.*, 2020). Table 3 contains a list of all software tool versions used, where appropriate.

**Table 3.** Software tools and versions used.

Software tool	Version	Source
BlobToolKit	3.3.10	Challis *et al*., 2020
freebayes	1.3.1-17-gaa2ace8	Garrison & Marth, 2012
Hifiasm	0.15.3	Cheng *et al*., 2021
HiGlass	1.11.6	Kerpedjiev *et al*., 2018
Long Ranger ALIGN	2.2.2	https://support.10xgenomics.com/genome-exome/software/pipelines/latest/advanced/other-pipelines
MitoHiFi	2.0	Uliano-Silva *et al*., 2021
PretextView	0.2	Harry, 2022
purge_dups	1.2.3	Guan *et al*., 2020
YaHS	1.0	Zhou *et al*., 2022

### Genome annotation

The Ensembl gene annotation system ( Aken *et al.*, 2016) was used to generate annotation for the *N. brevicollis* assembly (GCA_944738965.1). Annotation was created primarily through alignment of transcriptomic data to the genome, with gap filling via protein to-genome alignments of a select set of proteins from UniProt ( UniProt Consortium, 2019).

### Ethics/compliance issues

The materials that have contributed to this genome note have been supplied by a Darwin Tree of Life Partner. The submission of materials by a Darwin Tree of Life Partner is subject to the Darwin Tree of Life Project Sampling Code of Practice. By agreeing with and signing up to the Sampling Code of Practice, the Darwin Tree of Life Partner agrees they will meet the legal and ethical requirements and standards set out within this document in respect of all samples acquired for, and supplied to, the Darwin Tree of Life Project. Each transfer of samples is further undertaken according to a Research Collaboration Agreement or Material Transfer Agreement entered into by the Darwin Tree of Life Partner, Genome Research Limited (operating as the Wellcome Sanger Institute), and in some circumstances other Darwin Tree of Life collaborators.

## Data Availability

European Nucleotide Archive: *Nebria brevicollis*. Accession number PRJEB49041, https://identifiers.org/ena.embl/PRJEB49041 ( Wellcome Sanger Institute, 2022) The genome sequence is released openly for reuse. The *Nebria brevicollis* genome sequencing initiative is part of the Darwin Tree of Life (DToL) project. All raw sequence data and the assembly have been deposited in INSDC databases. Raw data and assembly accession identifiers are reported in Table 1.

## Author information

Members of the University of Oxford and Wytham Woods Genome Acquisition Lab are listed here: https://doi.org/10.5281/zenodo.4789928.

Members of the Darwin Tree of Life Barcoding collective are listed here: https://doi.org/10.5281/zenodo.4893703.

Members of the Wellcome Sanger Institute Tree of Life programme are listed here: https://doi.org/10.5281/zenodo.4783585.

Members of Wellcome Sanger Institute Scientific Operations: DNA Pipelines collective are listed here: https://doi.org/10.5281/zenodo.4790455.

Members of the Tree of Life Core Informatics collective are listed here: https://doi.org/10.5281/zenodo.5013541.

Members of the Darwin Tree of Life Consortium are listed here: https://doi.org/10.5281/zenodo.4783558.
