# Supplementary material for: The genome sequence of a ground beetle, Nebria brevicollis (Fabricius, 1792)
Source: Wellcome Open Res. 2023 Jan 12;8:20. [Version 1] doi: 10.12688/wellcomeopenres.18749.1 (PMC10521042; doi:10.12688/wellcomeopenres.18749.1)
Supplement: Peer review history

# Reviewer report: Reviewer response for version 1

**DOI:** 10.21956/wellcomeopenres.20789.r66358

**Published:** 2023-09-18

**Authors:** Sandro Patroca da Silva

**Affiliations:** 1 Evandro Chagas Institute, Ananindeua, Brazil

The research paper, titled "The Genome Sequence of *Nebria brevicollis* (Fabricius, 1792)," comprehensively elucidates the nuclear genome and mitogenome sequences of *Nebria brevicollis*, available in the public database. It meticulously outlines the methodology employed, providing a clear roadmap for potential replication and further exploration of this groundbreaking genomic study on this ground beetle species.

Are sufficient details of methods and materials provided to allow replication by others?

Yes

Is the rationale for creating the dataset(s) clearly described?

Yes

Are the datasets clearly presented in a useable and accessible format?

Yes

Are the protocols appropriate and is the work technically sound?

Yes

Reviewer Expertise:

Engaged in bioinformatic analysis encompassing viruses, metagenomics, and mitogenomes.

I confirm that I have read this submission and believe that I have an appropriate level of expertise to confirm that it is of an acceptable scientific standard.

# Reviewer report: Reviewer response for version 1

**DOI:** 10.21956/wellcomeopenres.20789.r54068

**Published:** 2023-01-23

**Authors:** Sean Schoville

**Affiliations:** 1 Department of Entomology, University of Wisconsin-Madison, Madison, WI, USA

Crowley and colleagues present the first genome sequence of *Nebria brevicollis*, an widespread and well studied ground beetle. The genome is an important contribution to our knowledge of beetle genomic diversity and the resources developed in the paper are of high quality. I found the paper to be well written and the graphics are nicely composed.

 Among the existing beetle genomes, this is one of the smaller assemblies and perhaps worth discussing. It is also of note that a previous estimate of chromosome number in *N. brevicollis* is widely different (N 30): Serrano, J., 1982 ^1^.

 Possible contamination: I was surprised, given previous genome reports, that no microbial contaminants/symbionts were found. However, the blobplot does have some unusual scatter. Perhaps a second approach, based on k-mer matching, might be helpful for a comprehensive check.

 There is a previously published mitochondrial genome for *N. brevicollis* (KT876906), perhaps a quick comparison would benefit readers:

- Linard B, Arribas P, Andújar C, Crampton‐Platt A, Vogler AP (2016) Lessons from genome skimming of arthropod‐preserving ethanol. *Molecular Ecology Resources* **16**, 1365-1377 ^2^.

Minor revisions:

- 2nd sentence of background: Typical convention is that " *Nebria*" should be spelled out if it starts a sentence, even if the full taxonomic name has been mentioned previously.

Are sufficient details of methods and materials provided to allow replication by others?

Yes

Is the rationale for creating the dataset(s) clearly described?

Yes

Are the datasets clearly presented in a useable and accessible format?

Yes

Are the protocols appropriate and is the work technically sound?

Yes

Reviewer Expertise:

Population genomics and comparative genomics of insects

I confirm that I have read this submission and believe that I have an appropriate level of expertise to confirm that it is of an acceptable scientific standard.

## References

[1] : New Chromosome Numbers of Spanish Caraboidea (Coleoptera, Adephaga). *Genética ibérica* .1982;34(63-82) :

[2] : Lessons from genome skimming of arthropod-preserving ethanol. *Mol Ecol Resour* .2016;16(6) : 10.1111/1755-0998.12539 1365-1377 10.1111/1755-0998.12539 27235167
